# An uncommon co-localization of rDNA 5S with major rDNA clusters in Callichthyidae (Siluriformes): a report case in *Corydoras carlae* Nijssen & Isbrücker, 1983

**DOI:** 10.3897/CompCytogen.v10i4.9507

**Published:** 2016-11-18

**Authors:** Rafael Henrique da Rocha, Lucas Baumgärtner, Leonardo Marcel Paiz, Vladimir Pavan Margarido, Carlos Alexandre Fernandes, Éder André Gubiani

**Affiliations:** 1 Universidade Estadual do Oeste do Paraná, Centro de Engenharias e Ciências Exatas, 85903-000 Toledo, Paraná, Brazil; 2 Universidade Estadual do Oeste do Paraná, Centro de Ciências Biológicas e da Saúde, 85819-110 Cascavel, Paraná, Brazil; 3 Universidade Estadual de Maringá, Centro de Ciências Biológicas, 87020-900 Maringá, Paraná, Brazil; 4 Universidade Estadual de Mato Grosso do Sul, Unidade Universitária de Mundo Novo, 79980-000 Mundo Novo, Mato Grosso do Sul, Brazil

**Keywords:** Ag-NORs, cytogenetic markers, ribosomal DNA, heterochromatin, karyotype

## Abstract

*Corydoras* Lacepède, 1803 is the most specious genus of Corydoradinae subfamily and many of its species are still unknown in relation to molecular cytogenetic markers. However, the diploid number and karyotypic formula were recorded for many species of this group. In current study, we provided the first cytogenetic information of *Corydoras carlae* Nijssen & Isbrücker, 1983, an endemic fish species from Iguassu River basin, Paraná State, Brazil. The individuals were collected in Florido River, a tributary of Iguassu River and analysed with respect to diploid number, heterochromatin distribution pattern, Ag-NORs and mapping of 5S and 18S ribosomal genes. The karyotype of this species comprises 46 chromosomes arranged in 22m+22sm+2st. The heterochromatin is distributed in centromeric and pericentromeric positions in most of the chromosomes, and also associated with NORs. The Ag-NORs were detected in the terminal position on the long arm of the metacentric pair 6. The double-FISH technique showed that 5S rDNA and 18S rDNA were co-localized in the terminal portion on the long arm of the metacentric pair 6. This condition of co-localization of ribosomal genes in *Corydoras carlae* seems to represent a marker for this species.

## Introduction

In higher eukaryotes, rDNA is organized into two distinct gene classes: major class (45S rDNA), which contains the genes that code for the 18S, 5.8S and 28S rRNAs, and the minor class (5S rDNA), which contains the genes that code for 5S rRNA. Fish species have been analyzed for 5S and 18S rDNA location in chromosomes using fluorescent *in situ* hybridization (FISH). The major rDNA sequences detected by FISH always coincided with silver-stained NORs (Ag-NORs) location, although in species with multiple Ag-NORs the number of markings was usually smaller than the regions detected by the DNA probes.

The most common condition in the karyotype of different fish groups is the positioning of ribosomal genes in different chromosome pairs (Galetti Jr. and Martins 2004). However, syntenic localization of the major rDNA clusters and the 5S sites were observed for the first time in the genus *Corydoras* Lacepède, 1803 (present study) and *Callichthys callichthys* (Linnaeus, 1758) (Konerat et al. 2014), the other integrant of the family Callichthyidae. In Loricariidae, *Kronichthys lacerta* (Nichols, 1919), *Isbrueckerichthys duseni* (Miranda Ribeiro, 1907), *Parotocinclus maculicauda* (Steindachner, 1877) and *Trichomycterus* sp. (Ziemniczak et al. 2012) also presented syntenic localization of ribosomal genes. Thus, the mapping of ribosomal genes has added important information about the chromosomal diversification in *Corydoras*, as in other groups of Siluriformes.

Callichthyidae is a family of the order Siluriformes widely distributed in Neotropical region, which has 215 valid species, divided in two subfamilies, Callichthyinae with 17 valid species and Corydoradinae with 198 valid species (Eschmeyer and Fong 2016). *Corydoras* is the most specious and cytogenetically studied genus of Corydoradinae, demonstrating different diploid numbers, which may vary from 2n = 40 chromosomes in *Corydoras nattereri* Steindachner, 1876 (Oliveira et al. 1990, 1993) to 2n = 134 chromosomes in *Corydoras aeneus* (Gill, 1858) (Turner et al. 1992).

Considering aspects related to number and morphology of chromosomes, as well as analysis of DNA content, Oliveira et al. (1992) and Shimabukuro-Dias et al. (2004) proposed the existence of five groups of species in *Corydoras*. However, the vast majority of studies in *Corydoras* is restricted to conventional analysis and little is known about location of the different types of rDNA, only in *Corydoras paleatus* (Jenyns, 1842) and *Corydoras ehrhardti* Steindachner, 1910 for 18S rDNA (Artoni et al. 2006) and *Corydoras britskii* (Nijssen & Isbrücker, 1983) for 18S and 5S rDNA (Takagui et al. 2014), making essential the development of studies with this approach to better understand the relationships between species of *Corydoras*.

Thus, the current paper presents the first cytogenetic description of *Corydoras carlae*, focusing on karyotype characterization, heterochromatin distribution pattern and location of 5S and 18S rDNA sites. Besides the new data for the species, this study also reveal for the first time the co-localization of 5S and major rDNA in Callichthyidae.

## Materials and methods

**Figure 1.**
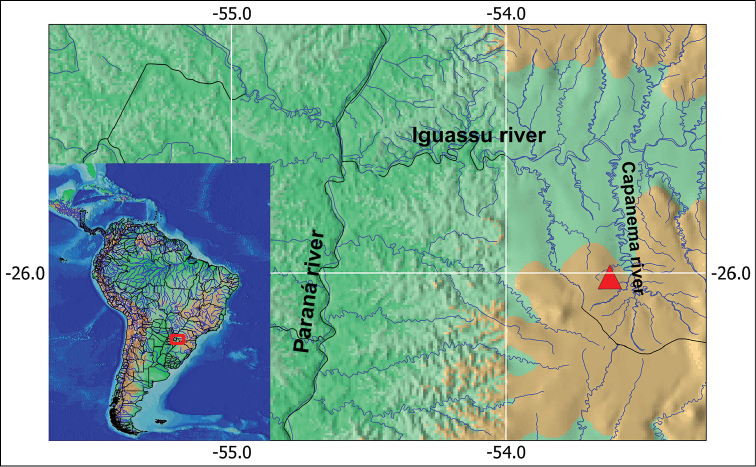
Localization of Florido River from the Iguassu River basin, where *Corydoras carlae* individuals were captured. Red triangle indicates the sampled point.

Ten individuals (four females and six males) of *Corydoras carlae* were sampled in the Florido River (26°00'32.60"S; 53°25'50.70"W), Paraná State, Brazil. A sub-tributary of left margin of Lower Iguassu River that flows into Capanema River, which flows immediately above of the Iguassu falls (Fig. 1). Voucher specimens were deposited in the fish collection of the Núcleo de Pesquisas em Limnologia Ictiologia e Aquicultura (NUPELIA), Universidade Estadual de Maringá, Paraná, Brazil, as *Corydoras carlae* (NUP 17885).

This study was carried out in strict accordance with the recommendations of the Guide for the Care and Use of Laboratory Animals, approved by the Committee on the Ethics of Animal Experiments of the Universidade Estadual do Oeste do Paraná (License Number: Protocol 13/09 – CEEAAP/Unioeste). Before the evisceration process, the individuals were anesthetized by an overdose of clove oil (Griffiths 2000). Metaphase chromosomes were obtained from anterior kidney cells using the air-drying technique (Bertollo et al. 1978). Analysis of the C-positive heterochromatin (C-bands) followed the basic procedure of Sumner (1972), with some minor adaptations (Lui et al. 2012). The NORs were detected by means of silver nitrate staining (Ag-NORs), according to Howell and Black (1980). The chromosomes were classified as metacentric (m), submetacentric (sm), and subtelocentric (st) according to their arm ratio (Levan et al. 1964). For the determination of the fundamental number (FN), or number of chromosome arms, the m, sm and st chromosomes were considered as bearing two arms and the acrocentric chromosomes only one arm.

The localization of the 5S and 18S rDNA sites in the chromosomes was performed using the fluorescence *in situ* hybridization (FISH) method (Pinkel et al. 1986) with modifications (Margarido and Moreira-Filho 2008), with probes obtained from the fish species *Leporinus elongatus* Valenciennes, 1850 (Martins and Galetti Jr 1999) and *Prochilodus argenteus* Spix & Agassiz, 1829 (Hatanaka and Galetti Jr 2004), respectively. The probes were labelled through nick translation, with digoxigenin-11-dUTP (5S rDNA) and biotin-16-dUTP (18S rDNA) (Roche). Detection and amplification of the hybridization signal were made using avidin-FITC and anti-avidin biotin (Sigma) for probes labelled with biotin, and anti-digoxigenin rhodamine (Roche) for probes labelled with digoxigenin. Slides were counterstained with DAPI (50 µg/mL) and analysed in epifluorescence microscope (Olympus BX61). The images were captured using the software DP controller (Media Cybernetics).

## Results

**Figure 2.**
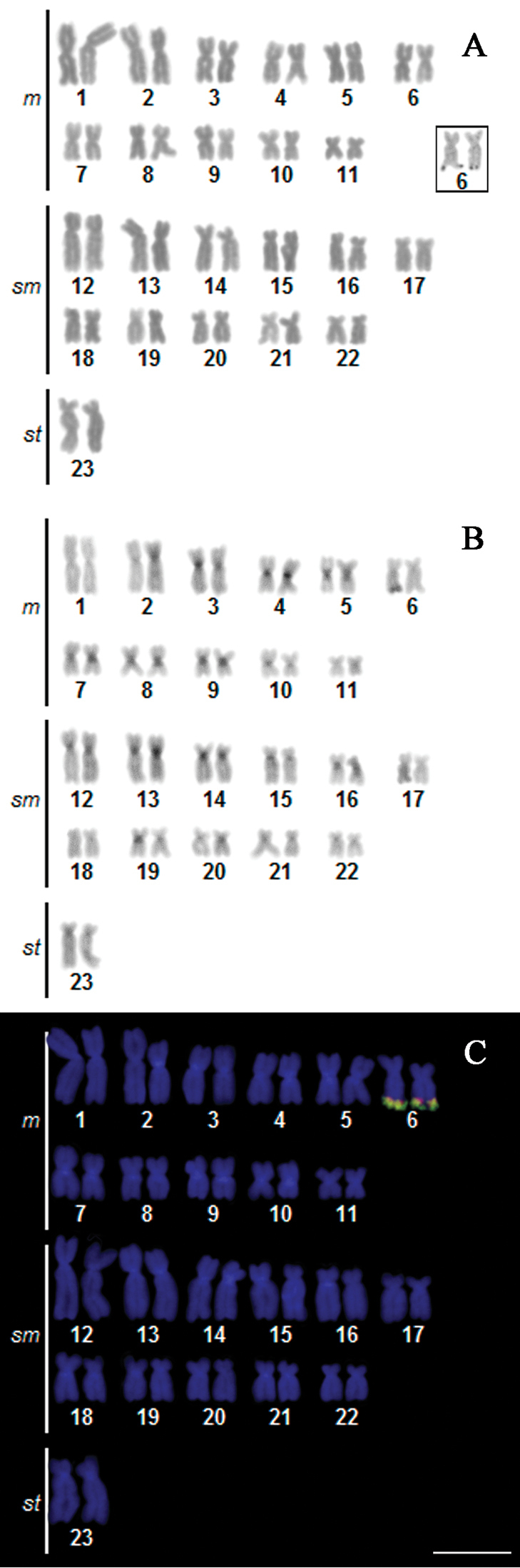
Karyotypes of *Corydoras carlae* stained with **a** Giemsa **b** C-banded and **c** after double FISH with 5S rDNA probes (red) and 18S rDNA (green). The NORs bearing chromosomes (pair 6) are boxed. Bar = 10 µm.

**Figure 3.**
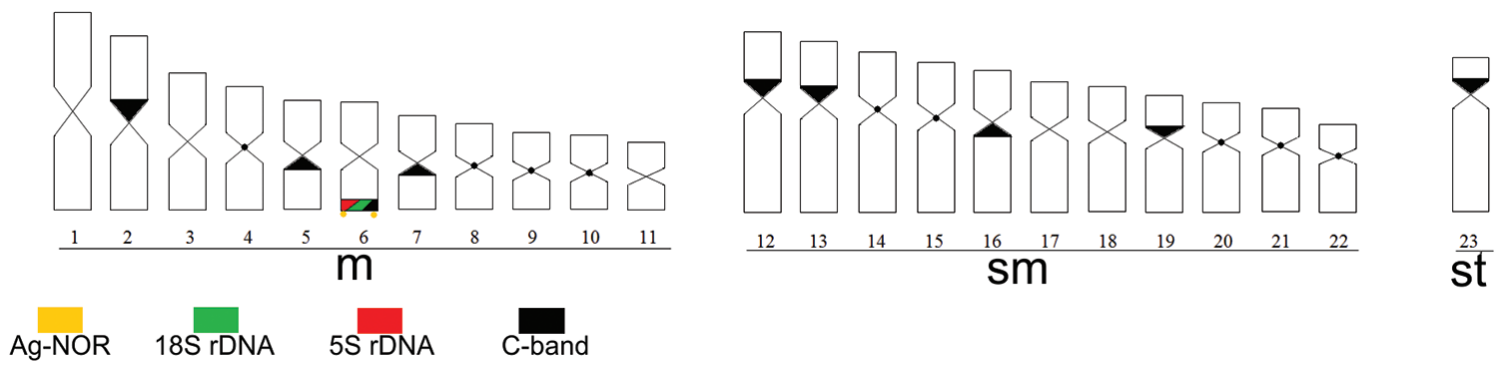
Ideogram of *Corydoras carlae*, showing the heterochromatin, Ag-NORs, 18S and 5S rDNA distribution pattern.

*Corydoras carlae* presented a modal diploid number of 46 chromosomes in males and females, and the karyotype contained 22 metacentric, 22 submetacentric and 2 subtelocentric chromosomes (22m+22sm+2st), yielding a FN of 92 in both sexes (Fig. 2a). The Ag-NORs was detected in the terminal position on the long arm of metacentric pair 6 (Box Fig. 2a). Positive C-band heterochromatins were detected in the centromeric and pericentromeric regions of nine and eight pairs, respectively, and coincident with the ribosomal sites (Fig. 2b). The double-FISH technique showed 5S rDNA cluster appears interspersed with 18S cistrons in the terminal portion of the long arm of pair 6 (Fig. 2c). Thus, featuring synteny and co-location of ribosomal genes in *Corydoras carlae*. The ideogram summarizes all markers on chromosomes of *Corydoras carlae* (Fig. 3).

## Discussion

Cytogenetic studies have classified the species of the genus *Corydoras* into five groups according to their karyotype composition (Oliveira et al. 1992, Shimabukuro-Dias et al. 2004). *Corydoras carlae* has been included in group 4 (2n = 40-52 chromosomes, with many metacentric and submetacentric chromosomes). Considering our results, three species of this group occurring in the Iguassu River basin were cytogenetically analyzed: *Corydoras carlae* (2n=46, 22m+22sm+2st), collected in the Lower Iguassu River; *Corydoras paleatus* (2n=44, 20m+24sm) collected in the Upper Iguassu River (Oliveira et al. 1993), and *Corydoras paleatus* and *Corydoras ehrhardti* (2n=44, 18m+26sm), collected in the Upper Tibagi River (Artoni et al. 2006).

Individuals of *Corydoras carlae* analyzed here probably do not co-occur with *Corydoras paleatus* from Upper Iguassu River, since the lower portion is characterized by numerous waterfalls which gave rise to several reservoirs (Baumgartner et al. 2012). Therefore, the geographic isolation of *Corydoras carlae* may have facilitated the establishment of this numerical and structural karyotypic variation, as also observed in different populations of *Glanidium ribeiroi* Haseman, 1911 along the Iguassu River basin (Lui et al. 2015). Thus, the lack of gene flow among *Corydoras* species in the Iguassu River basin could favor different changes in each sample, supposedly resulting in speciation.

The number and position of NORs in *Corydoras* species are quite variable and almost all information pertaining to the characterization of NORs in this species is based on silver nitrate impregnation (Table 1). These data show that most species have simple NORs located in the terminal position, as in the case of *Corydoras carlae*. However, not all species have this pattern, as in the case of *Corydoras simulatus* Weitzman & Nijssen, 1970 with interstitial NORs (Oliveira et al. 1992), as well as *Corydoras britskii* (Takagui et al. 2014), *Corydoras simulatus*, *Corydoras* sp. Galheiro river, *Corydoras flaveolus* Ihering, 1911 and *Corydoras metae* Eigenmann, 1914 (Oliveira et al. 1992), which exhibits a systems of multiple NORs. According to Oliveira and Gosztonyi (2000), the condition of simple Ag-NORs in terminal location is the possible basal condition for Siluriformes. Thus, *Corydoras carlae* and other species presenting simple Ag-NORs in terminal location seem to maintain this basal condition.

**Table 1.** Ag-NORs, major and minor ribosomal genes sites distribution in Callichthyidae. The 45S and 5S columns report the number of chromosomes bearing the cistrons and its location.

Family Callichthyidae	Locality	2n	Ag-NOR	45S	5S	Note	References
Subfamily Corydoradinae							
*Corydoras carlae*	Florido River/Paraná State, Brazil	46	simple	2, q terminal	2, q terminal	Synteny, Co-localization	Present study
*Corydoras britskii*	Miranda River/ Mato Grosso do Sul State, Brazil	90	multiple	3, p terminal	2 p, interstitial	Non-Synteny	Takagui et al. (2014)
*Corydoras paleatus*	Tibagi River/Paraná State, Brazil	44	simple	3, q terminal	-------	---------	Artoni et al. (2006)
*Corydoras ehrhardti*	Tibagi River/Paraná State, Brazil	44	simple	2, q terminal	-------	---------	Artoni et al. (2006)
*Corydoras sodalis*	from aquarium	74	simple	---------	---------	---------	Shimabukuro-Dias et al. 2004
*Corydoras arcuatus*	Tabatinga River/frontier Brazil and Peru	46	simple	---------	---------	---------	Oliveira et al. 1992
*Corydoras trilineatus*	Caripi River/Pará State, Brazil	46	simple	---------	---------	---------	Oliveira et al. 1992
*Corydoras schwartzi*	Negro River/Amazonas State, Brazil	46	simple	---------	---------	---------	Oliveira et al. 1992
Corydoras cf. simulatus	Colombia	62	simple	---------	---------	---------	Oliveira et al. 1992
*Corydoras* sp. *Caripi River*	Caripi River/Pará State, Brazil	60	simple	---------	---------	---------	Oliveira et al. 1992
*Corydoras reticulatus*	Negro River/Amazonas State, Brazil	74	simple	---------	---------	---------	Oliveira et al. 1992
Corydoras aff. punctatus Negro River	Negro River/Amazonas State, Brazil	102	simple	---------	---------	---------	Oliveira et al. 1992
*Corydoras simulatus*	Colombia	62	multiple	---------	---------	---------	Oliveira et al. 1992
*Corydoras* sp. Galheiro River	Galheiro River/Minas Gerais State, Brazil	84	multiple	---------	---------	---------	Oliveira et al. 1992
*Corydoras flaveolus*	Alambari River/São Paulo State, Brazil	58	multiple	---------	---------	---------	Oliveira et al. 1992
*Corydoras metae*	Colombia	92	multiple	---------	---------	---------	Oliveira et al. 1992
Subfamily Callichthyinae							
*Hoplosternum littorale*	Contas River/Bahia State, Brazil	60	simple	2, p terminal	4, p terminal	Non-Synteny	Almeida et al. (2012)
*Callichthys callichthys*	Paraná River/Paraná State, Brazil	56	simple	2-3, p terminal e interstitial	7-9, p interstitial and terminal	Synteny, Adjacent regions	Konerat et al. (2014)
*Hoplosternum littorale*	Coastal River/São Paulo State Brazil	60	simple	2, p terminal	4 p terminal	Non-Synteny	Pazza et al. (2005)
*Callichthys callichthys*	Contas River/Bahia State, Brazil	54	multiple	7, p terminal, 5, q terminal, 1 p interstitial	8-12, p interstitial and terminal	Non-Synteny	Almeida et al. (2013)
*Lepthoplosternum pectorale*	Paraná River/Paraná State, Brazil	64	multiple	10, p terminal; 2, q terminal	6, p terminal	Non-Synteny	Konerat et al. (2014)

Despite exhibiting wide variation on the diploid number, chromosome morphology and location of NORs, *Corydoras* species share a heterochromatin distribution pattern very similar, preferably centromeric and pericentomeric, and in most cases, associated to NORs. In *Corydoras carlae*, this pattern was also observed, with heterochromatic blocks also displayed in many chromosomes. *Corydoras britskii* from Miranda River also showed large amount of pericentromeric heterochromatin, but with terminal heterochromatic blocks (Takagui et al. 2014), which were not observed in this study.

The mapping of 18S rDNA and 5S rDNA are scarce in Callichthyidae, being known only for some species (Table 1). Concerning the genus *Corydoras*, *Corydoras carlae* exhibited only one chromosome pair bearing 18S rDNA sites, as well as *Corydoras ehrhardti* (Artoni et al. 2006), confirming the system of simple NORs evidenced by silver impregnation for both species. FISH with rDNA probes has helped detect the presence of inactive NORs, as in the case of *Corydoras paleatus* (Artoni et al. 2006), which presented multiple NORs sites after 18S-FISH, while the silver impregnation had detected only simple NORs. Thus, studies with 18S-FISH can be useful for better evaluating the pattern distribution of the NORs in *Corydoras*.

In *Corydoras*, data on the location and number of 5S rDNA cistrons had only been described for *Corydoras britskii*, for which was detected interstitially in a pair of subtelocentric chromosomes (Takagui et al. 2014). In *Corydoras carlae*, the 5S rDNA was displayed at terminal position on the long arm of the metacentric pair 6. The presence of one chromosome pair bearing 5S rDNA is a common feature in different families of Siluriformes (Swarça et al. 2009), although multiple loci of 5S rDNA have been observed in Callichthyinae (Table 1). Inter– and intra–individual numerical and position variations of 5S rDNA cistrons have been observed in Callichthyidae and seem to represent a species-specific marker.

Furthermore, 5S rDNA cluster appears interspersed with 18S cistrons, featuring synteny and co-location of ribosomal genes in *Corydoras carlae*. The synteny is an unusual feature in fish, and such condition could influence an unwanted translocation of 5S sequences within 45S clusters, which could probably occur if these clusters were maintained linked in the same chromosome area (Martins and Galetti Jr 1999). This may explain why most vertebrates have these sequences on different chromosomes. Interestingly, all the possible syntenic conditions have been found in fishes, both sets of genes in distinct and disjoint chromosomal regions, as observed in *Parodon nasus* Kner, 1859 cited as *Parodon tortuosus* (Vicente et al. 2001) and *Astyanax paranae* Eigenmann, 1914 cited as *Astyanax scabripinnis* (Mantovani et al. 2005), or in adjacent regions, as in *Triportheus nematurus* (Kner, 1858) (Diniz et al. 2009), *Mugil incilis* Hancock, 1830 (Hett et al. 2011), *Kronichthys lacerta*, *Isbrueckerichthys duseni*, *Parotocinclus maculicauda*, *Trichomycterus* sp. (Ziemniczak et al. 2012) and *Callichthys callichthys* (Konerat et al. 2014), or the 5S rDNA interspersed along the clusters of 45S rDNA (co-localization), as in *Astyanax altiparanae* Garutti & Britski, 2000, *Astyanax lacustris* (Lütken, 1875), *Astyanax fasciatus* (Cuvier, 1819), *Astyanax schubarti* Britski, 1964 and *Astyanax paranae* cited as *Astyanax scabripinnis* (Almeida-Toledo et al. 2002), *Solea senegalensis* Kaup, 1858 (Cross et al. 2006), *Bryconamericus* cf. *iheringii* (Piscor et al. 2013) and *Corydoras carlae* (present study).

Despite little studies about mapping of rDNA genes in Callichthyidae, the majority of the species share the condition of non-synteny between the 5S rDNA and 45S rDNA. Therefore, this condition of co-localization of ribosomal genes in *Corydoras carlae* seems to represent a marker for this species.
